# Gut microbiota-palmitoleic acid-interleukin-5 axis orchestrates benzene-induced hematopoietic toxicity

**DOI:** 10.1080/19490976.2024.2323227

**Published:** 2024-03-04

**Authors:** Lei Zhang, Ziyan Liu, Wei Zhang, Jingyu Wang, Huiwen Kang, Jiaru Jing, Lin Han, Ai Gao

**Affiliations:** a Department of Occupational Health and Environmental Health, School of Public Health, Capital Medical University, Beijing, China; b Department of Occupational Health and Environmental Health, School of Public Health, Binzhou Medical University, Yantai, China; c Beijing Key Laboratory of Environmental Toxicology, Capital Medical University, Beijing, China

**Keywords:** Benzene, hematotoxicity, gut microbiota, long-chain fatty acids, probiotics

## Abstract

Due to the annual increase in its production and consumption in occupational environments, the adverse blood outcomes caused by benzene are of concern. However, the mechanism of benzene-induced hematopoietic damage remains elusive. Here, we report that benzene exposure causes hematopoietic damage in a dose-dependent manner and is associated with disturbances in gut microbiota-long chain fatty acids (LCFAs)-inflammation axis. C57BL/6J mice exposed to benzene for 45 days were found to have a significant reduction in whole blood cells and the suppression of hematopoiesis, an increase in *Bacteroides acidifaciens* and a decrease in *Lactobacillus murinus*. Recipient mice transplanted with fecal microbiota from benzene-exposed mice showed potential for hematopoietic disruption, LCFAs, and interleukin-5 (IL-5) elevation. Abnormally elevated plasma LCFAs, especially palmitoleic acid (POA) exacerbated benzene-induced immune-inflammation and hematopoietic damage via carnitine palmitoyltransferase 2 (CPT2)-mediated disorder of fatty acid oxidation. Notably, oral administration of probiotics protects the mice against benzene-induced hematopoietic toxicity. In summary, our data reveal that the gut microbiota-POA-IL-5 axis is engaged in benzene-induced hematopoietic damage. Probiotics might be a promising candidate to prevent hematopoietic abnormalities from benzene exposure.

## 1. Introduction

Benzene is a well-established carcinogen for developing hematologic malignancies.^1^ Even less than 1 ppm of airborne benzene also can lead to non-carcinogenic hematotoxicity effects,^2,3^ such as reduced blood cell counts and suppressed hematopoietic capacity. Multipotent hematopoietic progenitor cells (MPPs) in the hematopoietic pool are directed toward a lineage outcome through cytokine-regulated cell fate decisions. Recent studies have reviewed the immunotoxic effects of benzene and highlighted the vital role of chronic inflammation and immune responses in the various adverse blood disorders associated with benzene.^4,5^ However, the role and mechanism of immunoinflammatory response in the pathogenesis of benzene-induced hematopoietic damage are largely unknown.

Evidence is mounting that gut microbial disorders may be critical pathogenesis for various diseases, including obesity, type 2 diabetes, nonalcoholic liver disease, and cardiovascular disease.^6^ Notably, the gut microbiota is significantly altered in cancer cell proliferation, hematopoietic stem cell (HSC) transplantation, and anti-cancer treatment.^7^ After two weeks of antibiotic treatment, reduced HSCs and MPPs were observed in the bone marrow (BM) of mice, accompanied by manifestations of anemia and white blood cells (WBC) reduction, which is attributed to the depletion of the gut microbiota due to antibiotic treatment, whereas this toxic effect can be reversed by fecal microbiota transplantation (FMT).^8^ Patients with acute lymphoblastic leukemia (ALL) tend to have both compositional and functional alterations in their metagenomes compared to people without ALL. The decreases of *Edwardsiella tarda* and *Prevotella maculosa* were found in ALL children, and these bacteria showed a significant positive correlation with IL-10.^9^ This evidence suggests that gut microbiota dysbiosis may profoundly influence hematologic progression by affecting specific cytokines of the immune system.

The molecular mechanisms responsible for the regulation of immune function and hematopoiesis by the gut microbiota are partially dependent on their metabolic production of compounds such as lipopolysaccharides, glucose, and fatty acids.^10^ Long-chain fatty acids (LCFAs) are classified as saturated or unsaturated fatty acids with 14–24 carbon atoms and play an indispensable role in membrane biosynthesis and the generation of signaling molecules. It has been reported that *Enterococcus faecalis* and its metabolite myristoleic acid (MOA) suppress obesity by increasing brown adipose tissue activity and fat beige formation.^11^ Oral administration of probiotic bacterial-derived 3-hydroxyoctadecaenoic acid was also proved to inhibit dextran sodium sulfate-induced colitis in mice.^12^ Oral administration of polyunsaturated fatty acids, such as docosahexanoic acid (DHA) and arachidonic acid, can promote hematopoiesis and platelet (PLT) production in mice.^13^ The heterogeneity of fatty acid carbon chain structures also predisposes them to perform different biological functions in the body. Saturated fatty acids promote inflammatory responses by rendering BM cells more responsive to pro-inflammatory stimuli.^14^ Several studies have linked hematologic malignancies to abnormal fatty acid metabolisms, such as fatty acid oxidation (FAO) and de novo synthesis.^15,16^ It is worth noting that the key enzyme for FAO, carnitine palmitoyltransferase 2 (CPT2), decreased by more than 85% in myelodysplastic syndromes patients, compared to healthy controls.^17^ Based on the above, we speculate that LCFAs may be critical mediators in the hematopoietic damage induced by benzene, and FAO is the critical metabolic pathway involved.

Benzene-induced hematopoietic damage characterized by a decrease in WBC or whole blood cells is a precursor to the development of hematologic malignancies. Until now, published research has only investigated correlations of benzene hematotoxicity with gut microbiota or metabolism,^18,19^ while systematic evidence of causation is lacking. In the present study, we reveal that the gut microbiota-LCFAs-IL-5 axis is involved in benzene-induced hematopoietic toxicity in mice. Benzene causes dysbiosis of the gut microbiota, particularly bacteria associated with LCFAs, by breaking the intestinal barrier. This alteration leads to abnormal elevation of plasma LCFAs, which exacerbates benzene-associated immunoinflammatory and hematopoietic damage through CPT2-mediated FAO dysfunction. The use of probiotics improved intestinal homeostasis in mice, which was beneficial for the recovery of hematopoietic function. Thus, our data provide new insights into the underlying mechanisms of benzene-induced hematotoxicity and highlight the potential protection of probiotics against the adverse blood outcomes associated with benzene.

## 2. Methods

### 2.1. Animal experiments

Male C57BL/6J mice (18–20 g, six weeks old) were obtained from Capital Medical University Laboratory Animal Center. All mice were housed in 3–5 per cage and maintained under a controlled temperature (22 ± 1°C), humidity (50 ± 5%), and lighting conditions (7:00 AM–7:00 PM).

Experiment 1: establishment of a mouse model of hematopoietic toxicity

To evaluate the dose effect of benzene exposure on hematopoietic toxicity in mice, all mice were divided into four groups as follows (*n* = 5/group): control (corn oil) and three experimental groups treated with the oil-benzene mixture (5, 25, 125 mg/kg•bw benzene). Five consecutive subcutaneous injections per week were performed for 45 days. The body weight of the mice was obtained once a week during benzene exposure. Detailed descriptions of the choice criteria for benzene doses had been described in previous studies.^18^ Because the most obvious changes in hematopoietic toxicity and fatty acid levels were observed in mice at a benzene dose of 125 mg/kg in the present study, the higher dose was used in subsequent intervention experiments to investigate the mechanism in depth.

Experiment 2: the effect of FMT on hematopoietic toxicity in mice

To determine the causal association between altered gut microbiota and benzene-induced hematopoietic damage, we performed FMT experiments with stools from donor mice treated with or without 125 mg/kg benzene (*n* = 8/group). Starting from the first day of exposure, fresh stools were collected from donor mice via metabolic cages and transferred to recipient mice matched for age and sex (*n* = 8/group). Briefly, the stools from control or benzene donors were dissolved in sterile saline (100 mg/mL) and adequately mixed by vortexing. The suspension was filtered with a 70 µm membrane and centrifuged at 800×g for 3 min (repeated two times). Finally, the supernatant was collected and delivered to recipient mice by oral gavage (100 µL per mouse). Recipient mice received a total of 33 microbiota transplants, five times per week for 45 days. Before FMT, recipient mice were fed antibiotics ad libitum in drinking water (ciprofloxacin, 0.2 g/L; metronidazole, 1 g/L; Sigma-Aldrich, USA) for five days to deplete the native microbiota as previously reported.^20,21^

Experiment 3: the effect of supplementation with MA and POA on hematopoietic toxicity in mice

Following a previous study,^22^ myristic acid (MA) and palmitoleic acid (POA) (Sigma-Aldrich, USA) were dispersed in 10% bovine serum albumin (BSA) solution using an ultrasound water bath to prepare a working solution at a concentration of 30 mg/mL, respectively. Ultimately, each mouse was orally gavaged 200 µL of MA or POA (300 mg/kg) to explore the effect of fatty acids on benzene-induced hematopoietic toxicity (*n* = 6/group). The exposure periods were the same as in experiment 1.

Experiment 4: the effects of CPT2 overexpression on hematopoietic toxicity in mice

To investigate the effects of CPT2 on benzene-induced hematopoietic toxicity, the CPT2 overexpression mice were constructed by tail vein injection of adeno-associated virus (AAV) vectors containing the genes for CPT2 (GeneChem Co., Ltd. Shanghai, China). Before benzene exposure, 200 µL of 2E + 11 vg/mouse AAV-GFP and AAV-CPT2 were delivered into mice in vivo using an AAV9 system (*n* = 10/group). After two weeks, benzene exposure treatment was performed according to the protocol of experiment 1. After 45 days, the coordinating effect of CPT2 overexpression on the benzene-induced hematopoietic damage was assessed by detecting CPT2 expression, blood cell counts, and HSC ratios.

Experiment 5: the effect of oral administration of probiotics on hematopoietic toxicity in mice

In the present study, we found that benzene exposure resulted in a downregulation of *Lactobacillus murinus* abundance, suggesting that benzene may affect intestinal homeostasis and promote hematopoietic toxicity by reducing the abundance of beneficial bacteria. Since there is no supplemental reagent for a single *Lactobacillus murinus*, we have used a commercial triple-activated bacteria reagent (Bifico, containing *Bifidobacterium longum*, *Lactobacillus acidophilus* and *Enterococcus faecalis*, Shanghai Xinyi Pharmaceutical Co.) for the intervention. This probiotic is currently in clinical use for the treatment of digestive disorders due to dysbiosis of the gut microbiota. To explore whether supplementation with probiotics could alleviate benzene-induced hematopoietic damage, control and 125 mg/kg benzene-exposed mice (*n* = 8/group) were treated with Bifico reagent. The reagent was suspended in sterile PBS and subsequently delivered to mice at a published dose of 4.2 g/kg^23^ (200 µL per mouse, active bacteria counts ≥ 1 × 10^7^ CFU). Five times a week for 45 days.

### 2.2. Hematopoietic damage phenotype

Femur histopathological analysis: Each group of three femur tissues was stripped of muscle and then immersed in 4% paraformaldehyde for 24 h. Before paraffin embedding, the femur was softened using a fresh decalcification solution in a controlled temperature shaker (25°C) for 30 days. Finally, a femur section with a thickness of 4 μm was prepared for hematoxylin and eosin (H&E) stain. Representative images of the full cross-section (×20 magnification) were obtained by using panoramic scanning (3DHISTECH, Hungary).

Routine blood test: After 45 days of benzene exposure, peripheral blood samples were collected by picking eyeballs under tribromoethanol anesthesia. Whole blood was used for the measurement of the WBC, red blood cells (RBC), hemoglobin (HGB) and PLT with a TEK-II mini automatic blood cell analyzer (Jiangxi Tekang Technology Ltd, China).

Hematopoietic stem cell ratio analysis: After being sacrificed, mice femurs and tibias were stripped and followed by BM cells were eluted with 5–10 mL of sterile PBS. Lin/Scal-1/c-Kit cells (LSK), long-term hematopoietic stem cells (LT-HSCs) and short-term HSCs (ST-HSCs) and MPPs are labeled by a panel of antibody markers (Biolegend, San Diego, California) for flow analysis. Detailed staining protocols are described in our published article.^18^ All data were uploaded into the FlowJo_V10 program for HSCs ratio analysis.

### 2.3. Intestinal damage phenotype

Intestinal histopathological analysis: After 45 days of exposure, three mice per group were randomly selected, weighed, and euthanized under tribromoethanol anesthesia. Afterward, fresh intestinal tissue was obtained by necropsy and immediately immersed in 4% paraformaldehyde for paraffin embedding. H&E and Alcian Blue-Periodic Acid Schiff (AB-PAS) stains were performed to examine intestinal epithelial damage and assess the barrier status of the mucus layer.

Intestinal permeability test: Mice fasted for 6 h were given 500 mg/kg•bw 4000 Da FITC-Dextran (Sigma-Aldrich, St. Louis, Missouri, USA) by oral gavage (125 mg/mL in PBS) as described in a previous study.^24^ After 4 h, 120–200 µL of blood was harvested via the tip of the tail vein, and the serum was isolated by low-speed centrifugation (3000 rpm, 4°C for 10 min). 25 µL of serum was diluted by adding 100 µL PBS and the OD value of each sample was measured by a full wavelength reader (Biotek Epoch2, USA) with an excitation wavelength of 485 nm and emission wavelength of 535 nm. Standard curves (0, 5, 10, 40, 80 mg/mL) were obtained by diluting FITC-dextran in non-treated serum diluted with PBS.

### 2.4. Bacterial 16s rDNA sequencing

After 45 days of exposure, the cecum contents of the mice were separated in an ultra-clean bench and transferred to 1.5 mL sterile tubes, immediately frozen in liquid nitrogen. The genomic DNA of mouse cecum contents was extracted by a PowerSoil® DNA isolation kit (MO BIO Laboratories, USA). A third-generation full-length (V1-V9) microbial diversity analysis based on the PacBio sequencing platform was performed to determine the relative abundance of differential gut microbes in different groups. The amplification of 16S rDNA bacterial genes was accomplished by the universal primers (27F: AGRGTTTGATYNTGGCTCAG; 1492 R: TASGGHTACCTTGTTASGACTT), and purified by magicpure size selection DNA beads. For the diversity analysis procedure, please refer to our previous study.^18,25^ Briefly, we corrected the original downstream subreads to obtain Circular Consensus Sequencing (CCS) sequences. To obtain high quality CCS sequences, lima (v1.7.0) software was utilized to identify CCS sequences of different samples by barcode sequences and remove chimeras. Sequences were clustered at 97% similarity level, and OTUs were filtered using 0.005% of the number of all sequenced sequences as a threshold. The Silva database (Releases 132, http://www.arb-silva.de/) was used to perform Genus annotation at a confidence threshold of 0.8, including kingdom, phylum, class, order, family, genus, and species. LefSe analysis was used to screen for differential microbes between groups. Linear discriminant analysis (LDA) was used to estimate the magnitude of the effect of abundance of each species on the differential effect.

### 2.5. Plasma metabolomic analysis

All plasma samples were maintained at −80°C until processed. The prepared samples were analyzed within 48 h after sample extraction and derivatization. Detailed sample pre-treatment procedures are described in the previous articles.^26^ Plasma metabolites were quantified by ultra-performance liquid chromatography coupled to a tandem mass spectrometry (UPLC-MS/MS) system (ACQUITY UPLC-Xevo TQ-S, Waters Corp., Milford, MA, USA). All UPLC experiments were performed on an ACQUITY UPLC BEH C_18_ analytical column (2.1 × 100 mm, 1.7 μm, Waters Corp) at 40°C. The mixture of water with 0.1% formic acid (A) and acetonitrile/IPA (70:30) (B) was used as the mobile phase and the gradient elution was set as follows: 0–1 min 5%B, 1–11 min a linear increase to 78%B, 11–16 min 100%B, 16–18 min a linear decrease to 5%B. The flow rate was set at 0.4 mL/min and the injection volume was 5 μL. Over 60 internal standards were added to the test samples in order to monitor analytical variations during the entire sample preparation and analysis processes. Raw data were processed using the iMAP platform (v1.0, Metabo-Profile, Shanghai, China) based on the m/z value and retention time of metabolite.^27^

### 2.6. Inflammatory cytokines analysis

Plasma samples were thawed on an ice bath and centrifuged at 10,000 rpm for 10 min. The supernatant was diluted 4-fold using a sample diluent. Finally, 50 μL of the diluted sample was prepared for the assay. Plasma cytokines were quantified by Bio-Plex MAGPIX System (Bio-Rad). Measurement of 12 cytokines (including IL-1α, IL-1β, IL-2, IL-3, IL-4, IL-5, IL-6, IL-9, IL-10, IL-12, IL-13, and IL-17A) in mouse plasma was performed using a multiplex assay kit (Bio-Plex).

### 2.7. Real-time quantitative PCR (qRT-PCR) analysis

BM cells: Total RNA from mouse BM cells was isolated with TRIzol reagent (Invitrogen, USA). Subsequently, 3 µg of RNA was reverse transcribed to cDNA using RevertAid First strand cDNA (Thermo Fisher Scientific, USA). The relative expressions of carnitine palmitoyltransferase 1A (CPT1A) and CPT2 were quantified by KAPA SYBR® FastMastermix kit (KAPABiosystems, USA). The reaction conditions for amplification were as follows: 95°C for 10 min, 40 cycles at 95°C for 15 s and 56°C for 30 s. The expression of target mRNA levels was normalized to β-actin content, and all experiments were performed in duplicate. The specific primer sequences as listed in Supplementary Table S1 were designed by PrimerBank, validated by the NCBI blast program, and synthesized by Sangon Biotech (Beijing, China).

Cecum content samples: Each cecum content sample was divided into 200 mg aliquots and stored at −80°C for total bacterial DNA extraction. Total bacterial DNA was extracted using a stool genomic DNA extraction kit (Solarbio Science & Technology Co., Ltd. Beijing, China) and was detected using a spectrophotometer (Epoch2t, BioTek Instruments, Inc. USA). Based on specific primers designed for the 16S rDNA gene, SYBR Green qualitative polymerase chain reaction (qPCR) master mix (BioRad, Hercules, CA) was used for amplification to detect the abundances of *Lactobacillus murinus*, *Akkermansia muciniphila*, *Bacteroides acidifaciens* and total bacteria in cecum content following a previous study.^21^ Finally, the abundance of target bacterial rDNA gene copy numbers relative to total bacterial rDNA gene copy numbers was calculated.

### 2.8. Immunohistochemistry (IHC)

Rehydrated sections of mouse femur tissue (*n* = 3/group) were washed with PBS (pH = 7.4), pretreated with 3% hydrogen peroxide for 25 min to inhibit endogenous peroxidase, washed again with PBS, and incubated in 3% BSA blocking solution for 30 min at room temperature. Sections were incubated with primary antibody (Abcam) against CPT2 (1:100, ab181114) at 4°C overnight. After washing, sections were incubated in a biotinylated secondary antibody (Servicebio Co. Ltd., Wuhan, China) for 50 min at room temperature and labeled with 3-diaminobenzidine. Finally, cell nuclei were re-stained using hematoxylin for 3 min and dehydrated to seal the slices for image acquisition. Semi-quantitative analysis of CPT2 expression was performed by Image Pro Plus version 6.0.

### 2.9. Western blot analysis

The total protein of 1 × 10^6^/mL BM cell of each mouse was extracted by whole cell lysis assay kit (KeyGen, Jiangsu, China) and quantified by the BCA protein assay kit (Dingguo Biotechnology Co., Ltd., China) at 562 nm. BM cell proteins were isolated on 10% sodium dodecyl sulfate-polyacrylamide gel electrophoresis (SDS-PAGE) and transferred to the polyvinylidene fluoride membrane. After blocking, the membranes were incubated overnight with an anti-CPT2 antibody (1:3000; Abcam, USA) and an anti-β-actin antibody (1:3000; Abcam, USA). Finally, bands were imaged by the chemiluminescence method (Merck Millipore, USA) and grayscale values were detected by Image J software.

### 2.10. Subject characteristics and sample collection

From December 2018 to February 2019, 76 healthy controls (office staff) and 86 benzene-exposed workers (paint sprayers) were recruited from the same auto repair shop in Beijing. The benzene exposure group was included according to the following criteria: age less than 50, benzene exposure for more than 2 years, no other history of occupational exposure related to benzene exposure, no history of long-term smoking or alcohol consumption, and no metabolic diseases. The control group was healthy individuals matched to the benzene exposure group by age and sex. The basic characteristics (Supplementary Table S3) and hematological indicators of the subjects were described in our previous study.^28^ In the present study, we obtained informed consent from all subjects, and the protocol was approved by the Ethics and Human Subject Committee of Capital Medical University (2017054).

3 mL of fasting venous blood was collected at the time of health physical examination in both groups of subjects. Plasma was obtained by centrifugation at 4°C for 10 min at 1500 g and stored at −80 for cytokine measurements. Finally, a total of 162 plasma samples were obtained for the measurement of cytokine levels.

### 2.11. Statistical analysis

All data analyses were performed using GraphPad Prism 8.3, Origin 2022b and SPSS 26.0 software, and all significance levels were set at 0.05 (two-tailed). The Benjamin-Hochberg (BH) method was used to control the false discovery rate (FDR), with FDR values less than 0.25 as the threshold for significance of differences.^29^ Differences between groups were examined for statistical significance using Student’s t-test or the Mann-Whitney U test. Correlation analysis between different indicators was performed by using the Spearman test.

## 3. Results

### 3.1. Benzene exposure caused inflammation and hematopoietic damage in mice

**Figure 1.**
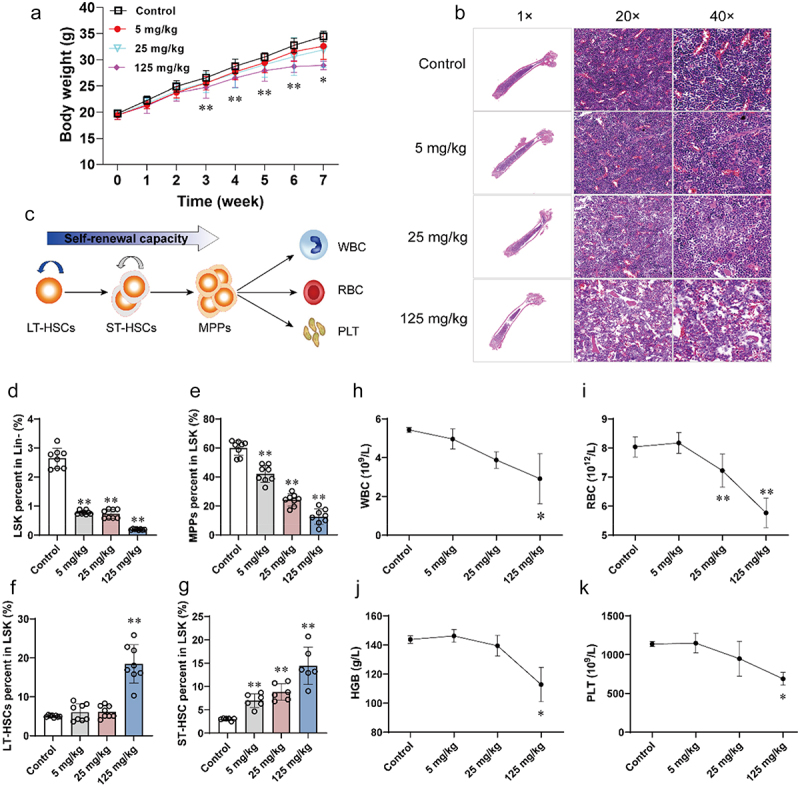
Alterations in body weight, and phenotype of hematopoietic damage in mice after 45 days of benzene exposure.

In this study, the male C57BL/6J mice were exposed to 5, 25, and 125 mg/kg of benzene for 45 days. After three weeks of exposure, body weight was found to be significantly reduced in benzene-exposed mice (Figure 1a). Since depression of BM hematopoiesis is the primary toxic effect of benzene, we first observed changes in femur pathology, HSC ratios, and peripheral blood cell counts in mice after benzene exposure. The pathological features of mouse femurs showed no significant alteration in BM hematopoietic phenotype between the control and 5 mg/kg benzene-exposed group. However, reduced HSCs and increased lipid droplets were found in 25 and 125 mg/kg benzene-exposed groups, which initially indicated that benzene causes hematopoietic damage in mice (Figure 1b). BM is an ordered environment for the renewal and differentiation of HSCs, which are responsible for the generation of all blood cells and immune cells (Figure 1c). Flow cytometry results revealed that 5 mg/kg of benzene already significantly disturbed the renewal and differentiation capacity of mouse HSCs, characterized by a decrease in LSK and MPPs and an increase in LT-HSCs and ST-HSCs by a dose-dependent manner (Figure 1d–g). Finally, we performed routine blood tests on the mice and a significant decrease in WBC, RBC, HGB, and PLT was found in 125 mg/kg benzene-exposed group (Figure 1h–k).

(a) Body weight trend in benzene-exposed and control mice (*n* = 5/group); (b) Representative pathological images magnified 1×, 20× and 40× of H&E staining of mouse femur sections (*n* = 3/group); (c) Schematic diagram of HSCs renewal and differentiation; (d – g) Alteration of HSCs (LSK, MPPs, LT-HSCs, and ST-HSCs) in BM cells of control and benzene-exposed mice by flow analysis (*n* = 8/group); (h – k) Alterations in blood cell counts (WBC, RBC, HGB, and PLT) in the control and benzene-exposed mice (*n* = 5/group). *P < .05, **P < .01, compared to the control group.

**Figure 2.**
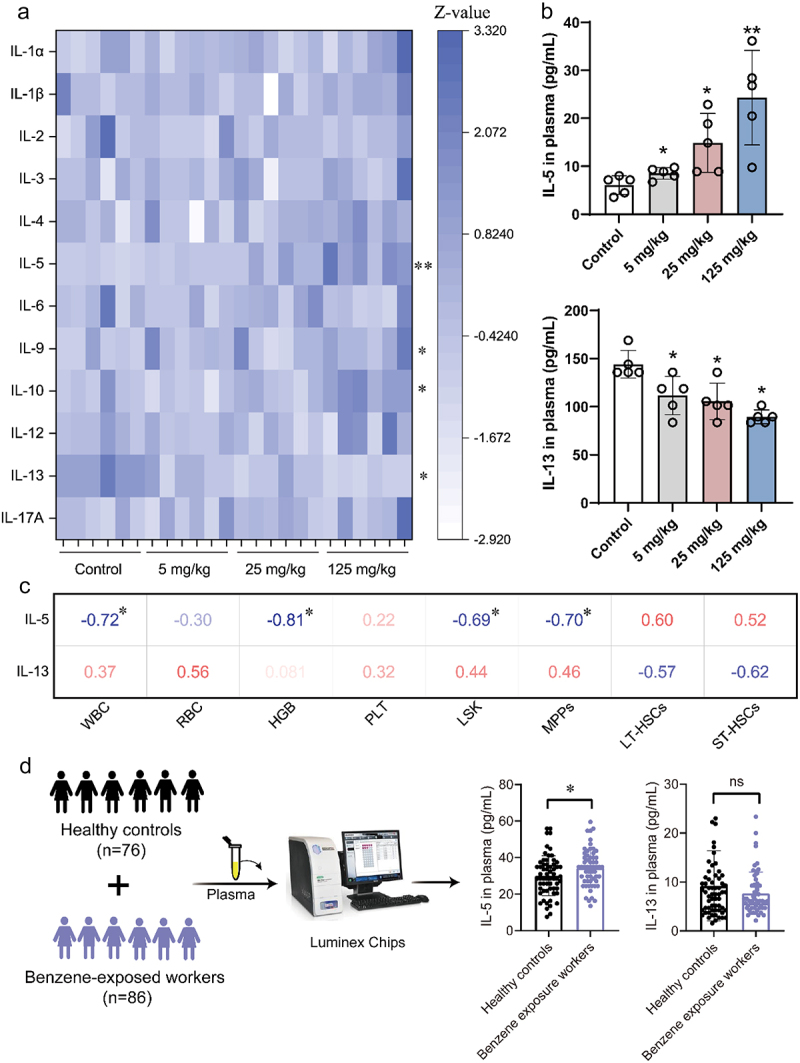
Absolute quantification of cytokines in plasma.

The immunoinflammatory response may be a pivotal process in benzene-induced hematotoxicity. Measurement of 12 cytokines in mouse plasma showed that four cytokines (IL-5, IL-9, IL-10, and IL-13) were significantly different between control and benzene-exposed groups (Figure 2a). Notably, pro-inflammatory factor IL-5 and anti-inflammatory factor IL-13 were significantly altered in a dose-dependent manner (Figure 2b). However, the correlation analysis only showed a significant negative correlation between IL-5 and WBC, HGB, LSK, and MPPs, and no relationship was found between IL-13 and hematopoietic damage (Figure 2c). We further validated the expression of these two cytokines in occupational benzene-exposed workers. As shown in Figure 2d, IL-5 expression was significantly upregulated in benzene-exposed workers compared with the healthy population, but there was no statistical difference in IL-13 expression. Taken together, the pro-inflammatory factor IL-5 might be a crucial factor in the modulation of benzene-induced hematopoietic disorders.

(a) The levels of inflammatory cytokines in mouse plasma following 45-day benzene exposure (*n* = 5/group); (b) Dose-dependent expression of IL-5 and IL-13 in mouse plasma (*n* = 5/group); (c) Correlation of plasma levels of IL-5 and IL-13 with hematopoietic-related indices in mice (Spearman analysis); (d) Plasma levels of IL-5 and IL-13 in healthy controls and benzene-exposed workers (*n* = 162). *P < .05, **P < .01, ns: *P* > .05, compared to the control group.

### 3.2. Benzene exposure disrupted the mucus barrier and microbial composition in mice

Gut microbiota is not only involved in intestinal diseases but also regulates the immune homeostasis of the BM hematopoietic environment through the gut-bone axis.^30^ In the present study, we first examined the pathological changes and permeability of the mouse intestine (Supplementary Figure S1). H&E staining results showed that benzene exposure caused atrophy of the intestinal mucosal epithelium and lymphocyte infiltration in the lamina propria of mice. AB-PAS staining was further used to check the mucin within the secretory granules of the cupped cells to assess the barrier status of the mucus layer. Compared to controls, the mucus layer of benzene-exposed mice was severely damaged, mainly manifested by the proliferation of cupular cells with a disorganized arrangement and reduced mucus area (Supplementary Figure S1a). In addition, the levels of FITC-Dextran in mouse serum were significantly increased after 45 days of exposure (Supplementary Figure S1b). This evidence suggested that benzene exposure disrupts the intestinal barrier and promotes intestinal permeability in mice.

**Figure 3.**
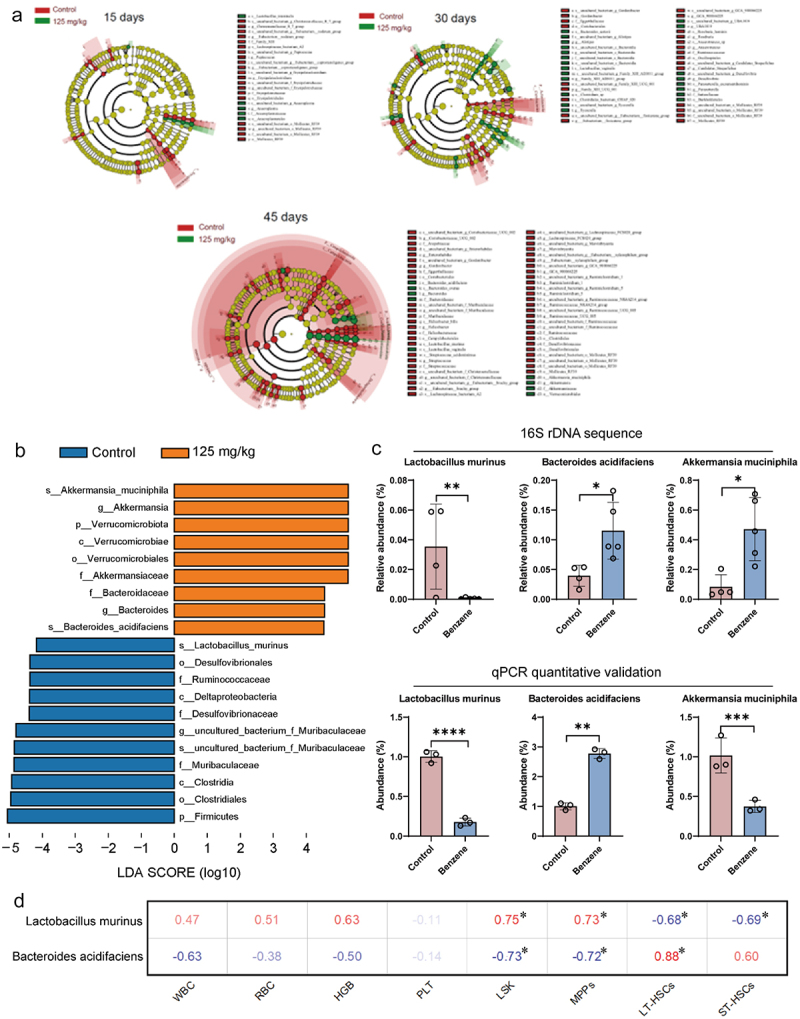
Altered gut microbiota composition in mice after benzene exposure.

The intestinal bacteria are mainly colonized in the mucus layer of the intestinal lumen. Benzene-induced damage to the intestinal mucus layer, but whether it further affects the homeostasis of the intestinal microbiota needs to be more investigated. Combining changes in microbial and WBC levels at different exposure times, our results preliminarily indicated that intestinal bacterial were more sensitive to benzene exposure compared to the distal bone marrow hematopoietic system, and benzene exposure affected more gut microbes in mice with increasing exposure time (Figure 3a and Supplementary Figure S2). In particular, at 45 days of benzene exposure, there was a 98.87% decrease of *Lactobacillus murinus* at the species level in the intestinal contents of the benzene-exposed mice compared to the control group. Conversely, the benzene-exposed group experienced a 5.69-fold increase in *Akkermansia muciniphila* and a 2.92-fold increase in *Bacteroides acidifaciens* at the species level. Furthermore, the qPCR results yielded the same trend in the changes of *Lactobacillus murinus* and *Bacteroides acidifaciens*, but with a conflicting *Akkermansia muciniphila* change (Figure 3b–c). Next, Spearman analysis revealed a striking correlation between differential microbes and hematopoietic damage (LSK, MPPs, LT-HSCs, and ST-HSCs) (Figure 3d and Supplementary Table S4), suggesting that gut microbiota might be involved in benzene-induced hematopoietic toxicity.

(a) Changes in the intestinal microbial profile of mice after 15, 30 and 45 days of benzene exposure; (b) LEfSe histogram of differential gut microbiota at 45 days of benzene exposure (LDA score > 4.0) (*n* = 5/group); (c) Gut microbiota with the greatest difference in relative abundance between the control and benzene-exposed groups were examined by 16S rDNA sequencing (*n* = 5/group) and qPCR quantification (*n* = 3/group); (d) Correlation analysis of differential gut microbiota and phenotype of hematopoietic damage. *P < .05, **P < .01, ***P < .001, compared to the control group.

### 3.3. Benzene exposure resulted in abnormal elevation of plasma fatty acids in mice

An underlying mechanism by which gut microbiota affect host energy metabolism is through the metabolites produced by their fermentation. In the present study, total quantitative targeted metabolomics was performed to find potential differential metabolites. As shown in the principal components analysis (PCA), partial least squares discriminant analysis (PLS-DA), and orthogonal partial least squares discriminant analysis (OPLS-DA), the altered metabolic profile of mice after 45 days of benzene exposure was significantly different from that of the control group (Supplementary Figure S3a – d). The orthogonal partial least squares discriminant analysis (OPLS-DA) model between control and benzene-exposed groups was 0.994 and 0.850 for the R2Y and Q2Y values, respectively (Supplementary Figure S3e). Based on this, VIP > 1 and *p* < .05 was chosen as the threshold for metabolite changes between the two groups.

**Figure 4.**
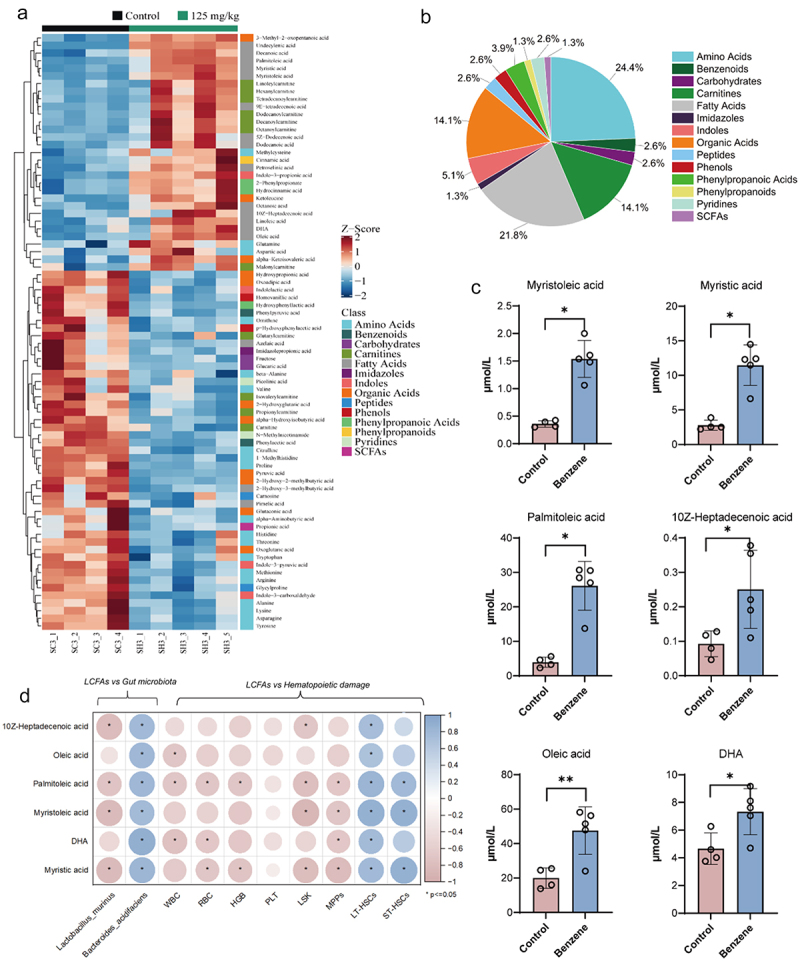
Altered plasma metabolite profiles in mice after 45 days of benzene exposure.

79 plasma metabolites were significantly altered in mice after benzene exposure for 45 days, with fatty acid classes accounting for 21.8% of all differential metabolites (Figure 4a, b). Detailed metabolite information was described in Supplementary Table S2. Of these, MOA, MA, POA, 10Z-Heptadecenoic acid (HA), Oleic acid (OA), and DHA were found to be consistently upregulated in benzene-exposed mice (Figure 4c). Further analysis also displayed a significant correlation between these identified LCFAs and the hematopoietic damage parameters (Figure 4d), and *Lactobacillus murinus* and *Bacteroides acidifaciens* were significantly associated with LCFAs (Figure 4d). These results recommend that LCFAs might act as messengers for gut microbiota in influencing host hematopoiesis.

(a) Heat map analysis of 79 identified plasma differential metabolites in control and benzene-exposed groups (*n* = 5/group); (b) Percentage distribution of differential metabolites; (c) Representative plasma LCFAs expression in the control and benzene-exposed groups (*n* = 5/group); (d) Associations of LCFAs with differential gut microbiota and hematopoietic damage parameters. *P < .05, **P < .01, compared to the control group.

### 3.4. Gut microbiota regulated benzene-induced hematopoietic damage by driving palmitoleic acid

**Figure 5.**
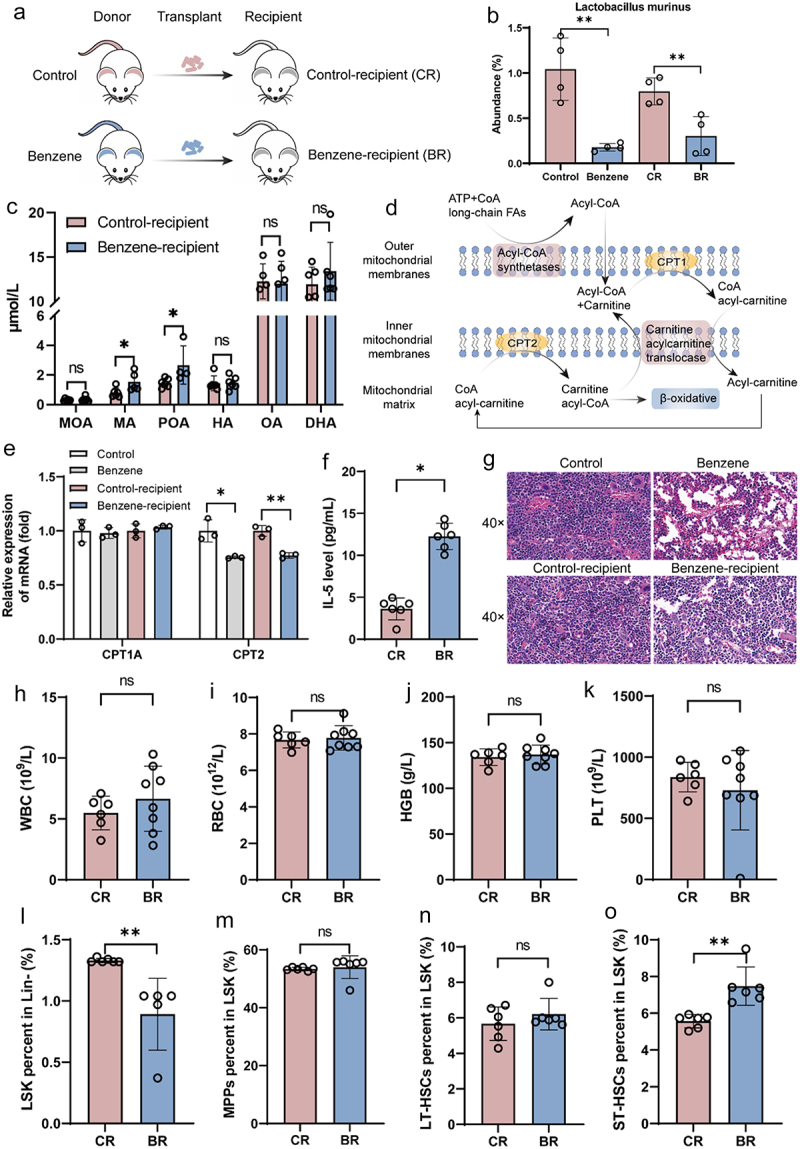
Gut microbiota contribute to benzene-induced hematopoietic damage by regulating fatty acid metabolism.

To confirm the relationship between the gut microbiota and LCFAs, fecal microbes from control or benzene-exposed mice were transplanted into recipient mice by oral gavage (Figure 5a). As expected, gut microbiota was colonized in recipient mice after 45 days of benzene exposure and the abundance of *Lactobacillus murinus* in benzene-recipient mice was significantly lower than in control-recipient mice (Figure 5b). In addition, plasma levels of six LCFAs remained elevated significantly in benzene-exposed mice. However, only MA and POA were significantly altered in benzene-recipient mice compared with control-recipient mice (Figure 5c). Fatty acids are involved in mitochondrial FAO as an energy source (Figure 5d). In this study, we found that the FAO pathway was significantly enriched (Supplementary Figure S3f). Furthermore, CPT2, a key rate-limiting enzyme for FAO, was significantly inhibited after benzene exposure, whereas CPT1A was not considerably altered (Figure 5e). Notably, after FMT, benzene-recipient mice possess similar hematopoietic damage characteristics to benzene-exposed mice, including altered pathology as well as decreased LSK and increased ST-HSCs (Figure 5g–o), accompanied by elevated pro-inflammatory factor IL-5 (Figure 5f). These results suggested that gut microbiota-derived MA and POA might promote benzene-induced immunoinflammatory and hematopoiesis by inhibiting CPT2-mediated FAO.

(a) Schematic diagram of fecal microbiota transplant experiment; (b) Abundance of Lactobacillus murinus in the intestinal contents of mice after fecal microbiota transplant was determined by qPCR (*n* = 4/group); (c) Alteration of LCFAs levels in recipient mice (*n* = 6/group); (d – e) Changes in critical rate-limiting enzyme for fatty acid oxidation (*n* = 3/group); (f) Alteration of plasma IL-5 levels in recipient mice (*n* = 6/group); (g – o) Alterations in BM pathology, peripheral blood counts (*n* = 8/group) and HSCs (*n* = 6/group) in recipient mice. *P < .05, **P < .01, ns: *P* > .05, compared to the control group.

### 3.5. Increased plasma palmitoleic acid promoted inflammation and hematopoietic damage in mice

**Figure 6.**
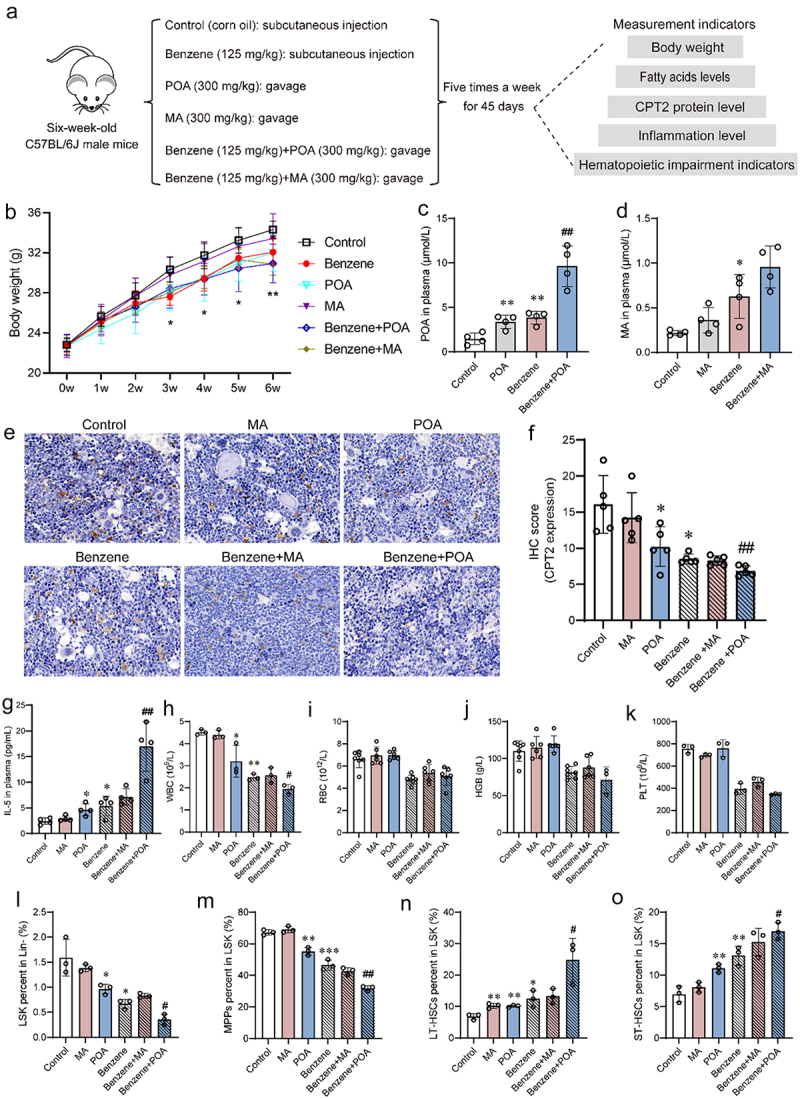
Elevated LCFAs promote benzene-induced hematopoietic toxicity.

To investigate the relationship between abnormal fatty acid changes and hematopoietic damage, control and benzene-exposed mice were supplemented with MA and POA by oral gavage for 45 days (Figure 6a). Starting from the third week, mice treated with benzene, benzene+MA, and benzene+POA showed a significant decrease in body weight compared to the control group (Figure 6b). After 45 days of exposure, plasma levels of POA and MA were higher in benzene+POA and benzene+MA groups compared to benzene-exposed group, but only the former group was statistically different (Figure 6c, d). In addition, supplementation of POA more significantly inhibited the expression of CPT2 in BM cells of benzene-exposed mice (Figure 6e, f). Plasma IL-5 levels increased 3.2-fold in mice exposed to benzene+POA compared to mice treated with benzene alone (Figure 6g). Notably, supplementation with POA, but not MA, partially exacerbated benzene exposure-induced hematopoiesis, mainly manifested by a decrease in WBC, LSK, and MPPs as well as an elevation in LT-HSCs and ST-HSCs (Figure 6h–o). The above results suggested that POA supplementation promoted benzene-induced immune inflammation and hematopoietic damage and that CPT2 might be a key molecule involved in this process.

(a) Schematic diagram of the fatty acid intervention experiment; (b) Effect of supplementation with MA and POA on body weight in mice (*n* = 6/group); (c-d) Plasma MA and POA levels in mice after 45 days of exposure (*n* = 4/group); (e-f) The protein expression levels of CPT2 were analyzed by IHC (×50 Magnification, Scale bar: 20 μm) (*n* = 5/group); (g) Alteration of plasma IL-5 in mice after LCFAs supplementation (*n* = 4/group); (h – k) Changes in the number of peripheral blood cells in mice after LCFAs supplementation (*n* = 6/group); (l – o) Changes in the proportion of HSCs in BM of mice after LCFAs supplementation (*n* = 3/group). *P < .05, **P < .01, ***P < .001, compared to the control group; ^#^*P* < .05, ^##^*P* < .01, compared to the benzene-exposure group.

### 3.6. CPT2 overexpression alleviated immunoinflammatory and hematopoietic damage

**Figure 7.**
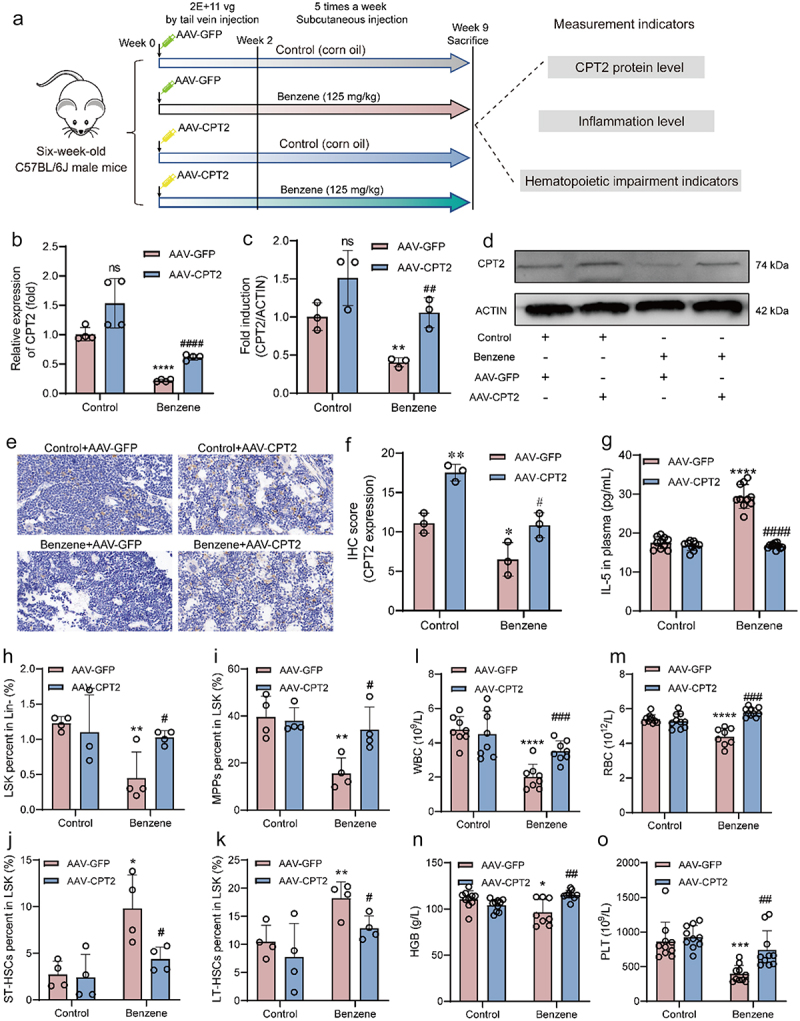
Effect of CPT2 overexpression on benzene-induced hematopoietic damage.

The role of CPT2 in hematopoietic damage was further investigated by establishing a CPT2 overexpression mouse model (Figure 7a). Combining the results of qPCR, western blotting, and immunohistochemistry, we found that the mRNA and protein levels of CPT2 were significantly increased in BM cells of mice treated with AAV-CPT2, indicating that the CPT2 overexpression mouse model was successfully constructed (Figure 7b–f). Compared to the benzene+AAV-GFP group, plasma IL-5 level was significantly downregulated in benzene-exposed mice administered AAV-CPT2 (Figure 7g), and the HSCs ratio and peripheral blood cell levels were also remarkably reversed (Figure 7h–o). This result suggested that targeting CPT2 intervention might be an effective strategy to combat benzene-induced hematopoietic toxicity.

(a) Schematic diagram of CPT2 overexpression experiment; (b) mRNA levels of CPT2 in mouse BM cells after injection of AAV-CPT2 (*n* = 4/group); (c-f) Protein levels of CPT2 in mouse BM cells were detected by western blotting assay and immunohistochemical assays (×50 Magnification, Scale bar: 20 μm) (*n* = 3/group); (g) CPT2 overexpression suppresses the abnormal elevation of plasma IL-5 in mice (*n* = 10/group); (h – k) CPT2 overexpression ameliorates benzene-induced abnormal HSCs ratios (*n* = 4/group); (l – o) CPT2 overexpression ameliorates benzene-induced peripheral blood cell decline (*n* = 10/group). *p < .05, **P < .01, ***P < .001, compared to the control+AAV-GFP group; ^#^*P* < .05, ^##^*P* < .01, compared to the benzene+AAV-GFP group.

### 3.7. Oral probiotics protect mice from benzene-induced hematopoietic damage

**Figure 8.**
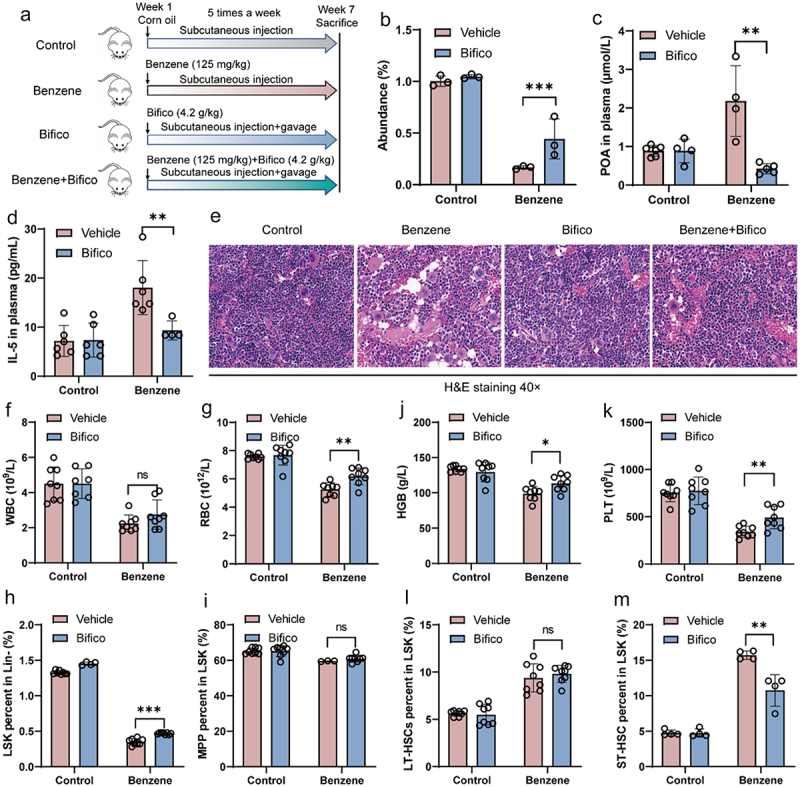
Probiotic supplementation protects the mice from benzene-induced hematopoietic damage.

The above results suggested that gut microbiota disruption was the primary driver of benzene-induced hematopoietic damage. Therefore, we tried to explore whether the intervention of intestinal homeostasis by probiotics supplementation could prevent hematopoietic toxicity. From the first day of exposure, a commercial probiotic agent (Bifico) was given orally to control and benzene-exposed mice (Figure 8a). qPCR results showed that probiotics supplementation significantly increased the abundance of *Lactobacillus murinus* in the intestinal contents of benzene-exposed mice (Figure 8b). In addition, we found that supplementation with probiotics significantly reversed the levels of plasma POA and IL-5 in benzene-exposed mice (Figure 8c, d). As expected, probiotic supplementation also protected mice from benzene-induced hematopoietic suppression and whole blood cell decline as evidenced by pathology, HSC ratio, and routine blood results (Figure 8e–m). Overall, our study revealed that probiotic treatment might potentially be an effective strategy to help prevent hematopoietic damage induced by benzene.

(a) Experimental design of oral probiotics; (b) Lactobacillus murinus colonization of mouse intestinal contents after oral supplementation of probiotics was analyzed by qPCR (*n* = 3/group); (c) POA levels in mouse plasma after oral administration of probiotics were detected by UPLC-MS/MS (*n* = 6/group); (d) IL-5 levels in mouse plasma were detected by ELISA after oral administration of probiotics (*n* = 6/group); (e) BM pathology in mice after oral administration of probiotics (×40 Magnification, Scale bar: 20 μm); (f – k) Changes in peripheral blood cell counts in mice after oral administration of probiotics (*n* = 8/group); (h – m) Changes in the proportion of BM HSCs in mice after oral administration of probiotics (*n* = 8/group). *P < .05, **P < .01, ns: *P* > .05.

## 4. Discussion

In the present study, our results strongly suggested that alterations in the gut microbiota-POA-IL-5 axis are at least one of the causes of benzene-induced hematopoietic toxicity. Probiotic supplementation might be a potential protection against adverse hematologic outcomes associated with benzene exposure.

Immunoinflammatory has been implicated as a potential regulator for benzene-induced hemotoxicity, although the mechanism remained unclear.^4,5^ In combination with our previous study,^18^ there was a significant increase in IL-5 levels in mouse plasma at either 30 or 45 days of benzene exposure. Notably, this result was also consistent in benzene-exposed workers in the present study. IL-5 is a member of the hematopoietic family of Th2 cytokines and acts on B cells and eosinophils to induce their growth and differentiation.^31^ Zhang et al. revealed that eosinophil-derived CCL-6 impairs HSC homeostasis.^32^ Therefore, we speculated that IL-5 mediated immune-inflammatory responses might be a pivotal factor in benzene-induced hematopoietic toxicity.

The intestine is home to trillions of active microorganisms that play an important role in the immune function and metabolism of the host.^33^ The microbial composition has been reported to be altered following benzene exposure. However, there are not entirely consistent changes in the bacterial community throughout the literature. Limited research has shown that the relative abundance of Actinobacteria at the phylum level and Helicobacter at the genus level in mice was significantly altered in hematological toxicity induced by 30 days of benzene exposure.^19^ However, the reduction of *Mollicutes RF39* at order level after 15 days of benzene exposure under the same exposure pathway raised more attention.^34^ In the immunotoxicity assessment of benzene, Karaulov et al. highlighted 45 days of benzene exposure as the most sensitive time point for benzene immunotoxicity.^35^ Therefore, we explored the alterations in the intestinal microbiota of mice after 45 days of benzene exposure. Compared to the control, the two microbes (*Lactobacillus murinus* and *Bacteroides acidifaciens* at the species) were dramatically and differentially altered after benzene exposure in the present study. Differences in the duration of exposure can influence the progression of hematotoxicity, and the immune response involved in it varies. This could be an explanation for the differences between studies.

Previous studies only established the links between gut microbiota and benzene-induced hematotoxicity via correlation analysis, while lacking an investigation of the causal relationship. Notably, given the FMT experiments reported in the present study, it was clear that dysbiosis of the gut microbiota is at least one of the causes of benzene-induced hematopoietic damage. We found that the relative abundance of *Bacteroides acidifaciens* increased and *Lactobacillus murinus* decreased significantly after benzene exposure, and the two microbes were strongly associated with hematopoietic damage. The mucus barrier is known as the primary defense against intestinal pathogens. Excessive numbers of *Bacteroides acidifaciens* can produce mucus-degrading enzymes that lead to a thinning of the mucus layer, allowing damage to epithelial cells by other harmful bacteria or antigens in the intestine.^36,37^ In addition, the relative abundance of *Lactobacillus murinus* was significantly decreased after benzene exposure. Therefore, an imbalance in the gut microbiota is probably a pivotal driver in promoting benzene-induced hematopoietic toxicity.

It is currently believed that active metabolites are powerful enforcers between gut microbiota and disease phenotypes.^38,39^ In particular, LCFAs (>12 carbon atoms) are engaged in critical functions such as receptor signaling, gene expression and modulation of energy homeostasis.^40^ Previous studies have shown that the production of 3-hydroxyoctadecenoic acid by bacteria might be one of the mechanisms associated with the anti-inflammation of probiotics.^12^ Both gut microbiota-driven MOA and 10-hydroxy-cis-12-octadecenoic acid showed excellent anti-obesity effects, which increases the potential for disease prevention and treatment from targeted metabolites.^11,41^ In addition, LCFAs are also essential energy providers, coming from FAO that produces more than twice as much ATP as glucose or amino acids.^16^ Our previous cross-sectional study revealed that plasma LCFAs were abnormally expressed in benzene-exposed workers and had a significant mediating effect in benzene-induced hematotoxicity.^28^ In the present study, we found that benzene exposure induced an abnormal increase in plasma levels of six LCFAs in mice, and FMT experiments validated POA to be a key metabolite for gut microbes to regulate benzene-induced hematopoietic damage. At the mechanism level, we found CPT2 expression in BM cells was significantly downregulated after benzene exposure. While CPT2 is closely related to FAO, mitochondrial dysfunction, and inflammation.^42,43^ Inhibition of CPT2 can increase the accumulation of acylcarnitine thereby establishing a lipid-rich tumor-promoting environment.^44^ Notably, several studies focusing on the downregulation of CPT2 expression the patients with blood disorders suggested its association with the development of hematopoietic damage,^17,45^ implicating the long-term effects of continued decline in CPT2. In the present study, supplementation with POA promoted immunoinflammatory and hematopoietic damage in benzene-exposed mice, while more significantly inhibiting CPT2 expression in BM cells. Notably, CPT2 overexpression could significantly reverse benzene-induced elevated pro-inflammatory factor IL-5 and hematopoietic damage. Taken together, these results indicated that a decline in *Lactobacillus murinus* is associated with abnormal elevation of plasma LCFAs. POA promotes benzene-induced hematopoietic toxicity by inhibiting CPT2-mediated FAO disorders.

The decrease in beneficial bacteria might be contributed to the exacerbated hematopoietic toxicity. Thus, we hypothesized that increasing the number of beneficial bacteria in mice through probiotic supplementation might help protect against benzene-induced hematopoietic toxicity. As an intestinal nutrient regulator, probiotics have played a protective role in ultrafine particle-induced colitis^21^ and perfluorobutane sulfonate-induced dysplasia.^46^ In addition, probiotic-based therapeutic strategies have been reported to help reduce the side effects of chemotherapy and eliminate multiple drug-resistant strains of bacteria in patients with hematologic malignancies.^47^ As expected, oral probiotics can restore abnormally elevated LCFAs, IL-5, and disturbed hematopoietic function. Given that our findings were revealed at the animal level, potential clinical trials testing the effects of probiotics are needed in the future to help protect the blood health of occupational benzene-exposed workers. This possibility may have public health significance because it is difficult to find more environmentally friendly alternatives to benzene in the current occupational environment.

Several limitations of our study also need to be noted. First, 16s rDNA sequencing lacks more biological information relative to the metagenome, and the potential functions of some bacteria closely related to benzene-induced hematopoietic toxicity need further investigation. Second, we have used antibiotics to deplete the gut microbes in the mouse background in the FMT experiments, but it is not yet equivalent to germ-free mice. Third, further efforts are required to explore the mechanism of action of probiotics against benzene-induced hematopoietic toxicity, which may facilitate the development of therapeutics. Fourth, although we found a statistically significant difference in serum IL-5 levels between healthy controls and benzene-exposed workers (*p* = .023), IL-5 levels were comparable between the two groups. This may be related to the small sample size. Nevertheless, this result suggested to some extent that IL-5 might play an important role in the process of benzene-induced hematopoietic toxicity. Therefore, to determine the relationship between IL-5 and benzene-induced hematopoietic toxicity, we need to validate it in a larger longitudinal cohort of occupational population.

## 5. Conclusion

Chronic benzene toxicity and benzene-induced leukemia are substantial health threats in benzene-exposed populations. Given the lack of understanding of the mechanisms involved, options for etiology-specific therapeutic and preventive strategies for individuals with benzene-induced adverse hematologic outcomes are limited. Our data help clarify the effect of the gut microbiota-POA-IL-5 axis on benzene-induced hematopoietic damage. Significantly, modulating intestinal homeostasis, such as treatment with probiotics, may help prevent or protect from hematopoietic damage caused by exposure to benzene.

## Supplementary Material

Revised Supplementary Materials clean.docx

## Data Availability

The microbial raw data reported in this paper have been deposited in the China National Center for Bioinformation (https://ngdc.cncb.ac.cn) and the accession numbers are PRJNA1068769.

## Abbreviations

ALL: Acute lymphoblastic leukemia
AB-PAS: Alcian Blue-Periodic Acid Schiff
BM: Bone marrow
CPT2: Carnitine palmitoyltransferase 2
DHA: Docosahexanoic acid
FAO: Fatty acid oxidation
FMT: Fecal microbiota transplantation
HSC: Hematopoietic stem cell
HGB: Hemoglobin
HA: 10Z-Heptadecenoic acid
H&E: Hematoxylin and eosin
IL-5: Interleukin-5
LCFAs: Long chain fatty acids
LSK: Lin/Scal-1/c-Kit cells
LT-HSCs: Long-term hematopoietic stem cells
LDA: Linear discriminant analysis
MPPs: Multipotent hematopoietic progenitor cells
MOA: Myristoleic acid
MA: Myristic acid
OA: Oleic acid
POA: Palmitoleic acid
PLT: Platelet
RBC: Red blood cells
ST-HSCs: Short-term HSCs
UPLC-MS/MS: Ultra-performance liquid chromatography coupled to a tandem mass spectrometry
WBC: White blood cells
