# Supplementary material for: Gut microbiota-palmitoleic acid-interleukin-5 axis orchestrates benzene-induced hematopoietic toxicity
Source: Gut Microbes. 2024 Mar 4;16(1):2323227. doi: 10.1080/19490976.2024.2323227 (PMC10913712; doi:10.1080/19490976.2024.2323227)
Supplement: Revised Supplementary Materials clean.docx [file KGMI_A_2323227_SM0085.docx]

**Supplementary material**

**Gut microbiota-palmitoleic acid-interleukin-5 axis orchestrates benzene-induced hematopoietic toxicity**

Lei Zhang^1,2^, Ziyan Liu^1^, Wei Zhang^1^, Jingyu Wang^1^, Huiwen Kang^1^, Jiaru Jing^1^, Lin Han^1^, Ai Gao^1,3*^

^1^ Department of Occupational Health and Environmental Health, School of Public Health, Capital Medical University, Beijing 100069, China

^2^ Department of Occupational Health and Environmental Health, School of Public Health and Management, Binzhou Medical University, Yantai 264003, China

^3^ Beijing Key Laboratory of Environmental Toxicology, Capital Medical University, Beijing 100069, China

**The supplementary material contains the following：**

Supplementary Table 1 The gene sequences of the specific primers being used in this study

Supplementary Table 2 General characteristics of subjects in two groups

Supplementary Table 3 79 identified differential metabolites in plasma between control and benzene-exposed mice

Supplementary Table 4 Correlation of key gut microbes and hematopoietic-related indicators

Supplementary Figure 1 Changes in intestinal damage and intestinal permeability in mice after benzene exposure.

Supplementary Figure 2 Changes in white blood cell levels in mice after 15, 30, and 45 days of benzene exposure.

Supplementary Figure 3 Multivariate statistical analysis of metabolic changes in plasma of control and benzene-exposed groups

| **Supplementary Table 1 The gene sequences of the specific primers being used in this study** | | |
| --- | --- | --- |
| Gene | Forward 5’-3’ | Reverse 5’-3’ |
| 16S | AGRGTTTGATYNTGGCTCAG | TASGGHTACCTTGTTASGACTT |
| Total bacteria | ACTCCTACGGGAGGCAGCAG | ATTACCGCGGCTGCTGG |
| Lactobacillus_murinus | AGCTAGTTGGTGGGGTAAAG | TAGGATTGTCAAAAGATGTC |
| Akkermansia muciniphila | CAGCACGTGAAGGTGGGGAC | CCTTGCGGTTGGCTTCAGAT |
| Bacteroides acidifaciens | GTATGGGATGGGGATGCGTT | CTGCCTCCCGTAGAGTTTGG |
| β-actin | CACCATGTACCCAGGCATTG | CCTGCTTGCTGATCCACATC |
| CPT1A | CTCCGCCTGAGCCATGAAG | CACCAGTGATGATGCCATTCT |
| CPT2 | CAGCACAGCATCGTACCCA | TCCCAATGCCGTTCTCAAAAT |

| **Supplementary Table 2 General characteristics of subjects in two groups** | | | | | |
| --- | --- | --- | --- | --- | --- |
| Characteristics | Control group (n=76) |  | Benzene-exposed group (n=86) | P value | FDR |
| Age (years) | 33 ± 8 |  | 30 ± 6 | <0.001^a^ |  |
| Gender (male), n (%) | 66 (86.8） |  | 80 (93.0) | 0.288^b^ |  |
| BMI (kg/m^2^) | 25.21 ± 5.17 |  | 26.16 ± 18.02 | 0.591^a^ |  |
| Smoking status, n (%) |  |  |  |  |  |
| Never | 26 (34.2) |  | 36 (41.9) | 0.345^b^ |  |
| Ever | 15 (19.7) |  | 20 (23.3) |  |  |
| Current | 35 (46.1) |  | 30 (34.9) |  |  |
| Alcohol consumption, n (%) |  |  |  |  |  |
| Never | 37 (48.7) |  | 44 (51.2) | 0.751^b^ |  |
| Ever | 38 (50.0) |  | 41 (47.7) |  |  |
| Current | 2 (2.6) |  | 2 (2.3) |  |  |
| Note: Kolmogorov-Smirnov test was used to confirm the normal distribution of raw data; Values are expressed as mean ± SD, median (IQR) or number of subjects (%). P^a^: Student's t-test; P^b^: chi-square test. | | | | |  |

| **Supplementary Table 3 79 identified differential metabolites in plasma between control and benzene-exposed mice** | | | | | |
| --- | --- | --- | --- | --- | --- |
| Class | Metabolite | P value | FDR | FC | log2FC |
| Amino Acids | 1-Methylhistidine | 1.92E-05 | 0.0020 | 0.6515 | -0.6181 |
| Amino Acids | Citrulline | 0.0003 | 0.0135 | 0.6918 | -0.5317 |
| Amino Acids | Methionine | 0.0013 | 0.0365 | 0.4731 | -1.0799 |
| Amino Acids | Tyrosine | 0.0063 | 0.0603 | 0.4834 | -1.0487 |
| Amino Acids | Threonine | 0.0084 | 0.0603 | 0.6989 | -0.5169 |
| Amino Acids | beta-Alanine | 0.0091 | 0.0603 | 0.6488 | -0.6241 |
| Amino Acids | Arginine | 0.01043 | 0.0603 | 0.6342 | -0.6569 |
| Amino Acids | Methylcysteine | 0.0119 | 0.0603 | 1.2708 | 0.3457 |
| Amino Acids | Ornithine | 0.01466 | 0.0603 | 0.5438 | -0.8788 |
| Amino Acids | Glutamine | 0.0159 | 0.0603 | 1.0949 | 0.1308 |
| Amino Acids | Asparagine | 0.0159 | 0.0603 | 0.5982 | -0.7414 |
| Amino Acids | alpha-Aminobutyric acid | 0.0159 | 0.0603 | 0.7642 | -0.3879 |
| Amino Acids | Proline | 0.0159 | 0.0603 | 0.5306 | -0.9142 |
| Amino Acids | Lysine | 0.0202 | 0.0717 | 0.7056 | -0.5030 |
| Amino Acids | Tryptophan | 0.0218 | 0.0734 | 0.8122 | -0.3001 |
| Amino Acids | Alanine | 0.0235 | 0.0768 | 0.6516 | -0.6179 |
| Amino Acids | Valine | 0.0393 | 0.1094 | 0.7550 | -0.4055 |
| Amino Acids | Histidine | 0.0457 | 0.1257 | 0.8304 | -0.2682 |
| Amino Acids | Aspartic acid | 0.0486 | 0.1286 | 1.4311 | 0.5171 |
| Benzenoids | Phenylacetic acid | 0.0001 | 0.0070 | 0.2315 | -2.1111 |
| Benzenoids | Phenylpyruvic acid | 0.0222 | 0.0737 | 0.5367 | -0.8978 |
| Carbohydrates | Glucaric acid | 0.0159 | 0.0603 | 0.5168 | -0.9523 |
| Carbohydrates | Tartaric acid | 0.0159 | 0.0603 | 0.9555 | -0.0657 |
| Carbohydrates | Fructose | 0.0357 | 0.1035 | 0.5658 | -0.8215 |
| Carnitines | Carnitine | 0.0015 | 0.0365 | 0.6224 | -0.6840 |
| Carnitines | Propionylcarnitine | 0.0058 | 0.0603 | 0.5990 | -0.7394 |
| Carnitines | Isovalerylcarnitine | 0.0128 | 0.0603 | 0.6268 | -0.6739 |
| Carnitines | Hexanylcarnitine | 0.0159 | 0.0603 | 3.1731 | 1.6659 |
| Carnitines | Octanoylcarnitine | 0.0159 | 0.0603 | 1.7048 | 0.7696 |
| Carnitines | Decanoylcarnitine | 0.0159 | 0.0603 | 1.2720 | 0.3471 |
| Carnitines | Dodecanoylcarnitine | 0.0159 | 0.0603 | 1.8642 | 0.8986 |
| Carnitines | Tetradecanoylcarnitine | 0.0163 | 0.0609 | 3.8011 | 1.9264 |
| Carnitines | Glutarylcarnitine | 0.0208 | 0.0723 | 0.6596 | -0.6004 |
| Carnitines | Malonylcarnitine | 0.0275 | 0.0869 | 1.5297 | 0.6133 |
| Carnitines | Linoleylcarnitine | 0.0377 | 0.1065 | 1.9537 | 0.9662 |
| Fatty Acids | Decanoic acid | 0.0032 | 0.0472 | 1.6087 | 0.6859 |
| Fatty Acids | Petroselinic acid | 0.0038 | 0.0528 | 2.6799 | 1.4222 |
| Fatty Acids | Octanoic acid | 0.0070 | 0.0603 | 2.8279 | 1.4998 |
| Fatty Acids | Oleic acid | 0.0077 | 0.0603 | 2.3782 | 1.2499 |
| Fatty Acids | Dodecanoic acid | 0.0091 | 0.0603 | 8.2642 | 3.0469 |
| Fatty Acids | Undecylenic acid | 0.0151 | 0.0603 | 10.7970 | 3.4326 |
| Fatty Acids | Azelaic acid | 0.0159 | 0.0603 | 0.5813 | -0.7827 |
| Fatty Acids | 5Z-Dodecenoic acid | 0.0159 | 0.0603 | 1.4800 | 0.5656 |
| Fatty Acids | Myristoleic acid | 0.0159 | 0.0603 | 4.3366 | 2.1166 |
| Fatty Acids | 9E-tetradecenoic acid | 0.0159 | 0.0603 | 3.7406 | 1.9033 |
| Fatty Acids | Myristic acid | 0.0159 | 0.0603 | 4.8769 | 2.2860 |
| Fatty Acids | Palmitoleic acid | 0.0159 | 0.0603 | 7.1995 | 2.8479 |
| Fatty Acids | Pimelic acid | 0.0194 | 0.0697 | 0.6896 | -0.5362 |
| Fatty Acids | 2-Hydroxy-3-methylbutyric acid | 0.0214 | 0.0732 | 0.7258 | -0.4624 |
| Fatty Acids | DHA | 0.0250 | 0.0804 | 1.5720 | 0.6526 |
| Fatty Acids | 10Z-Heptadecenoic acid | 0.0325 | 0.0986 | 2.7005 | 1.4332 |
| Fatty Acids | Linoleic acid | 0.0353 | 0.1035 | 1.5620 | 0.6434 |
| Imidazoles | Imidazolepropionic acid | 0.0159 | 0.0603 | 0.7036 | -0.5071 |
| Indoles | Indole-3-propionic acid | 0.0003 | 0.0108 | 4.1755 | 2.0620 |
| Indoles | Indole-3-pyruvic acid | 0.0019 | 0.0365 | 0.4953 | -1.0135 |
| Indoles | Indole-3-carboxaldehyde | 0.0159 | 0.0603 | 0.5023 | -0.9934 |
| Indoles | Indolelactic acid | 0.0169 | 0.0620 | 0.6235 | -0.6815 |
| Organic Acids | 3-Methyl-2-oxopentanoic acid | 1.11E-05 | 0.0020 | 2.5294 | 1.3388 |
| Organic Acids | Oxoadipic acid | 0.0001 | 0.0070 | 0.3913 | -1.3535 |
| Organic Acids | 2-Hydroxy-2-methylbutyric acid | 0.0019 | 0.0365 | 0.4339 | -1.2047 |
| Organic Acids | 2-Hydroxyglutaric acid | 0.0024 | 0.0400 | 0.6089 | -0.7157 |
| Organic Acids | Ketoleucine | 0.0040 | 0.0528 | 2.3760 | 1.2485 |
| Organic Acids | alpha-Hydroxyisobutyric acid | 0.0053 | 0.0603 | 0.2355 | -2.0863 |
| Organic Acids | alpha-Ketoisovaleric acid | 0.0054 | 0.0603 | 1.6803 | 0.7487 |
| Organic Acids | Hydroxypropionic acid | 0.0064 | 0.0603 | 0.6147 | -0.7020 |
| Organic Acids | Pyruvic acid | 0.0159 | 0.0603 | 0.6575 | -0.6049 |
| Organic Acids | Oxoglutaric acid | 0.0374 | 0.1065 | 0.6573 | -0.6054 |
| Organic Acids | Glutaconic acid | 0.0483 | 0.1286 | 0.5620 | -0.8314 |
| Peptides | Glycylproline | 0.0159 | 0.0603 | 0.9482 | -0.0767 |
| Peptides | Carnosine | 0.0326 | 0.0986 | 0.6566 | -0.6068 |
| Phenols | Homovanillic acid | 0.0076 | 0.0603 | 0.4853 | -1.0430 |
| Phenols | p-Hydroxyphenylacetic acid | 0.0469 | 0.1274 | 0.3837 | -1.3820 |
| Phenylpropanoic Acids | Hydrocinnamic acid | 0.0019 | 0.0365 | 2.7849 | 1.4776 |
| Phenylpropanoic Acids | Hydroxyphenyllactic acid | 0.0159 | 0.0603 | 0.4468 | -1.1621 |
| Phenylpropanoic Acids | 2-Phenylpropionate | 0.01587 | 0.0603 | 2.1841 | 1.1271 |
| Phenylpropanoids | Cinnamic acid | 0.0099 | 0.0603 | 3.9087 | 1.9667 |
| Pyridines | N-Methylnicotinamide | 0.0025 | 0.0400 | 0.1992 | -2.3279 |
| Pyridines | Picolinic acid | 0.0331 | 0.0988 | 0.4446 | -1.1695 |
| SCFAs | Propionic acid | 0.0318 | 0.0986 | 0.8828 | -0.1798 |
| P < 0.05 means the difference is statistically significant; The Benjamin-Hochberg (BH) method was used to control the false discovery rate (FDR), FDR-value < 0.25 was used as a significant level for the multiple comparison correction; Fold change (FC) means the ratio of metabolite expression in the benzene exposed group to the control group. | | | | | |

| **Supplementary Table 4 Correlation of key gut microbes and hematopoietic-related indicators** | | | | | | | | | |
| --- | --- | --- | --- | --- | --- | --- | --- | --- | --- |
| Bacterial | Statistical indicators | WBC | RBC | HGB | PLT | LSK | MPP | LT-HSC | ST-HSC |
| Lactobacillus murinus | r | 0.466 | 0.508 | 0.632 | -0.111 | 0.753* | 0.735* | -0.683* | -0.690* |
|  | P | 0.206 | 0.163 | 0.068 | 0.777 | 0.019 | 0.024 | 0.043 | 0.04 |
|  | FDR | 0.235 | 0.217 | 0.109 | 0.777 | 0.086 | 0.086 | 0.086 | 0.086 |
| Bacteroides acidifaciens | r | -0.630 | -0.375 | -0.500 | -0.139 | -0.733* | -0.724* | 0.875** | 0.599 |
|  | P | 0.069 | 0.320 | 0.170 | 0.722 | 0.025 | 0.027 | 0.002 | 0.088 |
|  | FDR | 0.138 | 0.366 | 0.227 | 0.722 | 0.072 | 0.072 | 0.016 | 0.141 |
| r: correlation coefficient; P: Original unadjusted P-value; FDR: Adjusted p-value by Benjamini-Hochberg method; * P< 0.05, ** P < 0.01. | | | | | | | | | |


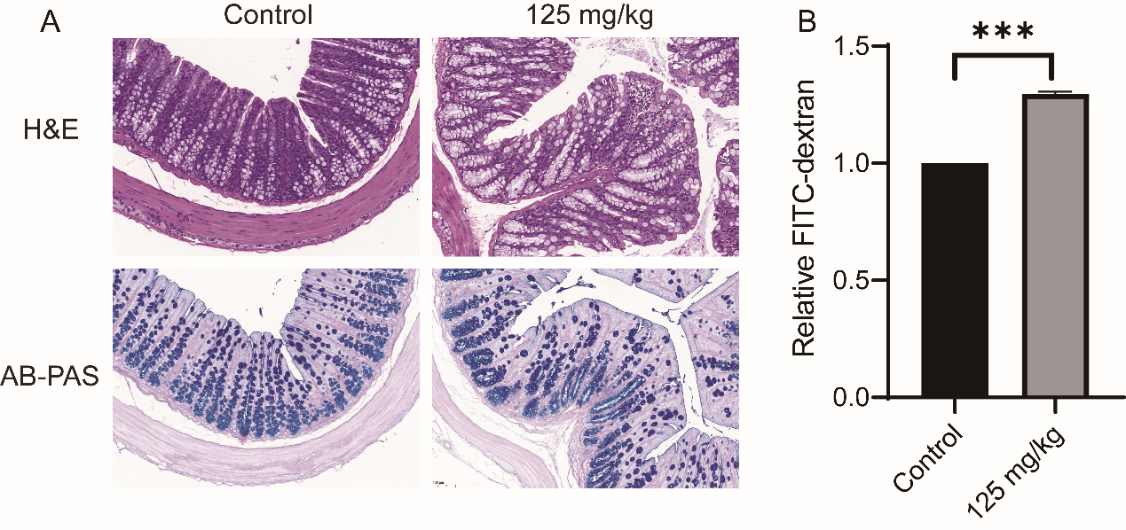


**Supplementary Figure 1 Changes in intestinal damage and intestinal permeability in mice after benzene exposure.** (A) Representative images magnified 15× of H&E and AB-PAS staining; (B) 4000 Da FITC-dextran serum levels assayed 4 h later. *** P < 0.001.

**Supplementary Figure 2**  Changes in white blood cell levels in mice after 15, 30, and 45 days of benzene exposure. Values are presented as means±SD. *P<0.05 indicates a statistically significant difference when compared to the control.


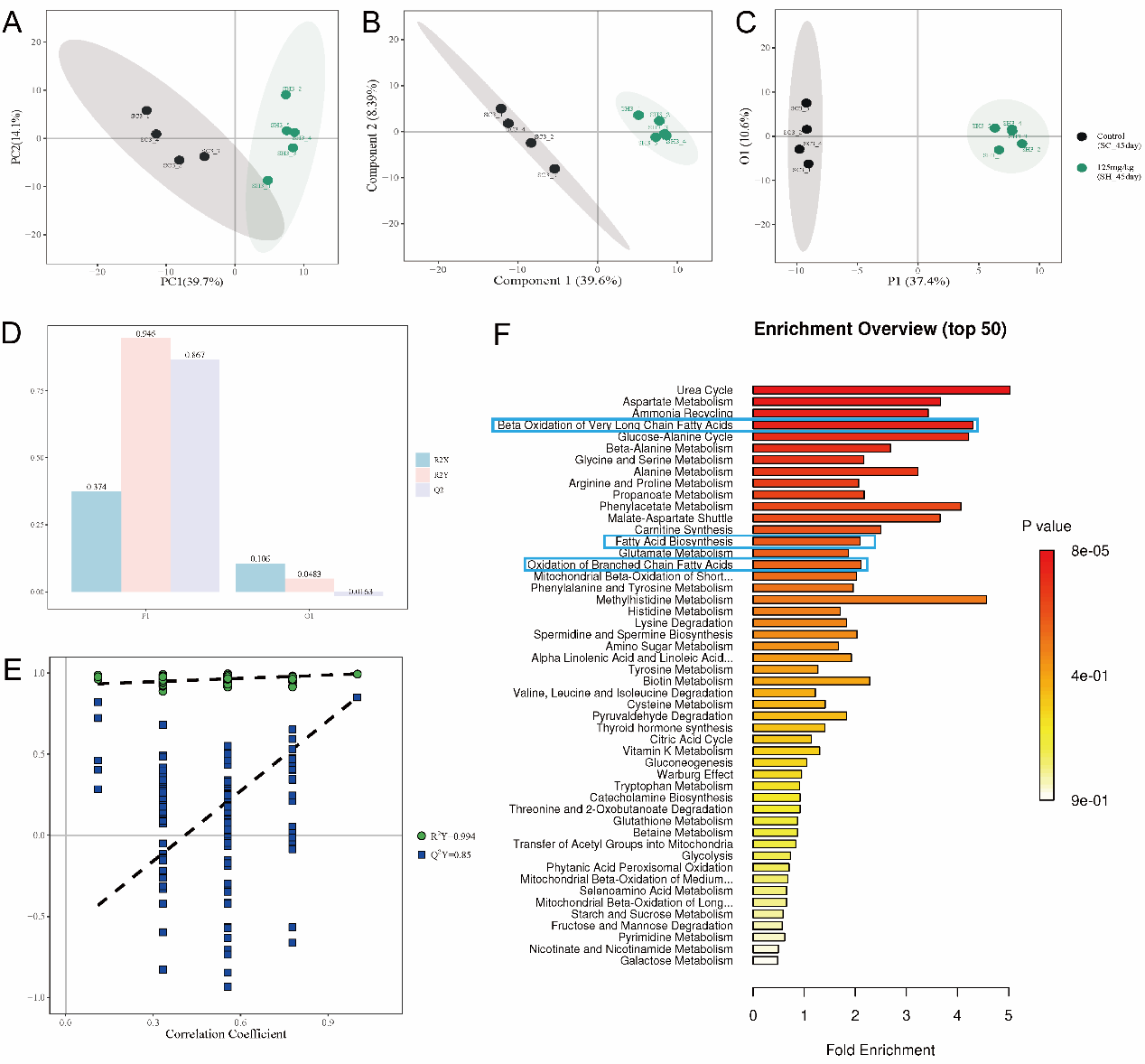


**Supplementary Figure 3 Multivariate statistical analysis of metabolic changes in plasma of control and benzene-exposed groups**. (A) PCA analysis; (B) PLS-DA analysis; (C-D) OPLS-DA analysis; (E) Validation plot of the model obtained from 1000 permutation tests; (F) Pathway enrichment analysis by KEGG.
